# Multiple Electrohydrodynamic Effects on the Morphology and Running Behavior of Tiny Liquid Metal Motors

**DOI:** 10.3390/mi9040192

**Published:** 2018-04-18

**Authors:** Yue Sun, Shuo Xu, Sicong Tan, Jing Liu

**Affiliations:** 1 Beijing Key Lab of Cryo-Biomedical Engineering and Key Lab of Cryogenics, Technical Institute of Physics and Chemistry, Chinese Academy of Sciences, Beijing 100190, China; sunyue161@mails.ucas.ac.cn (Y.S.); xushuo15@mails.ucas.ac.cn (S.X.); tansicong@mail.ipc.ac.cn (S.T.); 2 School of Future Technology, University of Chinese Academy of Sciences, Beijing 100039, China; 3 Department of Biomedical Engineering, School of Medicine, Tsinghua University, Beijing 100084, China

**Keywords:** liquid metal, electrical field, self-propulsion, droplet motor

## Abstract

Minimized motors can harvest different types of energy and transfer them into kinetic power to carry out complex operations, such as targeted drug delivery, health care, sensing and so on. In recent years, the liquid metal motor is emerging as a very promising tiny machine. This work is dedicated to investigate the motion characteristics of self-powered liquid metal droplet machines under external electric field, after engulfing a small amount of aluminum. Two new non-dimensional parameters, named Ä and Ö, are put forward for the first time to evaluate the ratio of the forces resulting from the electric field to the fluidic viscous force and the ratio of the friction force to the fluidic viscous force. Forces exerted on liquid metal droplets, the viscosity between the droplet and the surrounding fluid, the pressure difference on both ends, the friction between the bottom of the droplet and the sink base, and bubble propulsion force are evaluated and estimated regarding whether they are impetus or resistance. Effects of electric field intensity, droplet size, solution concentration and surface roughness etc. on the morphology and running behavior of such tiny liquid metal motors are clarified in detail. This work sheds light on the moving mechanism of the liquid metal droplet in aqueous solutions, preparing for more precise and complicated control of liquid metal soft machines.

## 1. Introduction

Minimized motors retain the property of their macroscale counterparts on harvesting different types of energy namely thermal [1,2], electrical [3,4], magnetic [5,6,7], chemical [8,9,10], ultrasound [11,12], light [13,14] power and so on, transferring into kinetic energy to carry out complex operations. Liquid metal, typically Gallium-based alloys, inherited with favorable fluidity, high thermal and electrical conductivity, large surface tension, low toxicity, and other excellent physical and chemical properties, has attracted huge interest across various fields, like chip cooling [15], nerve connection [16], drug delivery [17], flexible electronics [18], soft-bodied robot [19,20], micro pump [21], etc. Tiny liquid metal motors can be driven by the electric field, allowing its biocompatibility superior to those fueled by traditional toxic solution. Locomotion and morphology of room temperature liquid metal (RTLM) in multiphase fluidic environment and its precise control remain major challenges in the researches and applications of this promising material. Scattered into small droplets and provided with aluminum, autonomous macroscopic Brownian self-propulsion phenomenon of the tiny liquid metal motors can be observed in alkaline solutions [22]. These self-fueled motors can exhibit biomimetic behaviors without the involvement of external energy [7]. In the light of previous studies, a new robotic concept, Transient State Machine, was also put forward to describe machines that set off free morphological transition for different purposes [20].

Intensive studies have been performed to get command of the motion of liquid metal. Facts prove that liquid metal displays featured behaviors when subjected to electrical field, magnetic field [23,24], graphite substrate [25], ultraviolet-irradiation [26] and electrolyte properties like pH or ionic concentration gradients [27]. Among these, the mechanism of electric field-induced chemical locomotion of conduction objects [3] is more lucid than other factors. Tang et al. [28] had studied liquid metal droplets coated with nanoparticles, namely ‘liquid metal marbles’, which can be actuated upon the application of electric fields. In addition, they investigated the chaotic advection actuating [29] and pumping [21] performance of the millimeter scale liquid metal droplet under alternating current signal. Yang et al. [30] developed the liquid metal pump in milli/centi-meter scale aided with AC, and demonstrated its bulk and surface motions. Tan et al. revealed the electrical control and stimulation of the moving direction and the velocity of the tiny Al-Ga-In motors [31] as well as the magnetic restriction of their self-propulsion range, termed as the magnetic trap effect [24]. Tang et al. [32] described the usage of modest voltage to direct the continuous flow of liquid metal towards single or multiple directions simultaneously in micro-channels, which could work as a valve. Zhang et al. [33] demonstrated that a self-propelled motor with a nickel cap can be steered by magnetic or electric field, representing a reliable manner for drug delivery. In general, the effect of external fields on the liquid metal seems to be miraculous, especially when the liquid metal is fed with aluminum.

Based on previous studies of liquid metal-aqueous solution multiphase flow, this work focuses on the dynamic behaviors of liquid metal droplets after engulfing a small amount of aluminum, under the effect of electric field. Two non-dimensional parameters are put forward to evaluate the forces exerted on the droplet. Electric field intensity, droplet size, solution concentration and surface roughness are considered crucial factors to affect the morphology and running behavior of tiny liquid metal motors and various comparative experiments are performed. In the process of running, the liquid metal motor is subjected to the viscos dragging between the droplet and the surrounding fluid, the pressure difference on both ends, the friction between the bottom of the droplet and the sink base, and bubble propulsion force. Whether these forces propel or hinder the movement of liquid metal motors are discussed, revealing the moving mechanism of liquid metal in aqueous solutions.

## 2. Materials and Methods

Liquid metal used in this experiment is GaIn_10_, which contains 90% Gallium (Shanxi Zhaofeng Gallium Co., Ltd., Quanyang, China) and 10% Indium (Zhuzhou Smelter Group Co., Ltd., Zhuzhou, China) in weight. GaIn_10_ and Al are mixed in the ratio of 99:1 by weight. The liquid metal droplets are placed in a 12 cm × 1 cm × 1 cm open-top rectangular glass channel filled with sodium hydroxide solution. Two graphite rods are fixed at both terminals of the channel with a distance of 12 cm. A WYK-605 DC stabilized power supply (Dongfang Group East Co., Ltd., Dongguan, China) is configured to set up an adjustable electrical field. A high speed camera (Canon XF-305, Tokyo, Japan) is mounted above the channel to capture the motion. All the experiments were conducted under room temperature. To figure out the morphology and running behavior of the liquid metal droplet under different conditions, we investigate its performance with varied voltages, droplet sizes, solution concentrations and the friction force.

## 3. Results and Discussion

### 3.1. Mechanism Analysis

**Figure 1:**
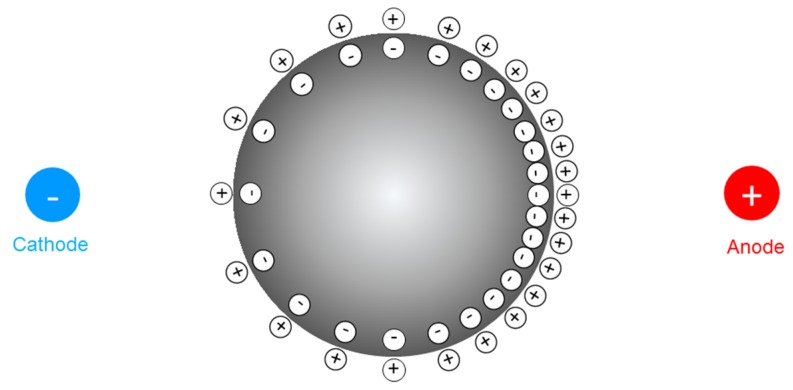
Surface charge redistribution of a liquid metal droplet in the electric field.

As the GaIn-Al droplet is placed in a channel filled with alkaline solution, a slow chemical reaction occurs between the alloy and the surrounding solution, producing gallates, [Ga(OH)_4_]^−^, resulting in a negative charge aggregation on the liquid metal droplet surface. The negatively charged surface attracts positive ions of the solution and a nearly homogeneous diffuse layer, electrical double layer (EDL) [34,35] is formed, which can be modeled as a charged capacitor. As noted, the GaIn-Al alloy possesses high conductivity, the potential through the droplet can be reckoned as uniform. In contrast, the aqueous electrical resistance is not neglected. Once a parallel electric field is implemented at both terminals, a potential gradient is generated along the channel. Thus, the potential difference of the EDL alters along the droplet surface, engendering the charge redistribution on the surface as Figure 1 depicts.

The electrical double layer on the surface affects the surface tension between the liquid metal and the solution, which can be described as Lippmann’s equation:

(1) $$\gamma =\gamma_{0}-\frac{1}{2}c{\left(V-V_{0}\right)}^{2}$$

where γ is the surface tension; $\gamma_{0}$ is the maximum surface tension when *V* = 0; *c* is the capacitance per unit area of the EDL; *V* is the electrode potential; *V*_0_ is the potential of zero charge. 

Supposing that there is no external electrical field, the EDL is initially charged by $q_{0}$, and the initial voltage of the capacitor is $V_{0}=q_{0}/c$. The voltage on the left side of the droplet is $V_{L}=V-\Delta \varphi_{r}/2$, and on the right side $V_{R}=V+\Delta \varphi_{r}/2$, where $\Delta \varphi_{r}$ is the potential difference of the external electrical field, φ is the potential in the EDL. It can be inferred that the surface tension on the left side of the liquid metal droplet is larger than that on the right side. Learning from the Young–Laplace’s equation, the pressure stress on the two sides of the droplet can be exhibited as:

(2) $$\Delta S=\frac{2\Delta \gamma }{R}=\frac{2q\Delta \varphi }{R}$$

**Figure 2:**
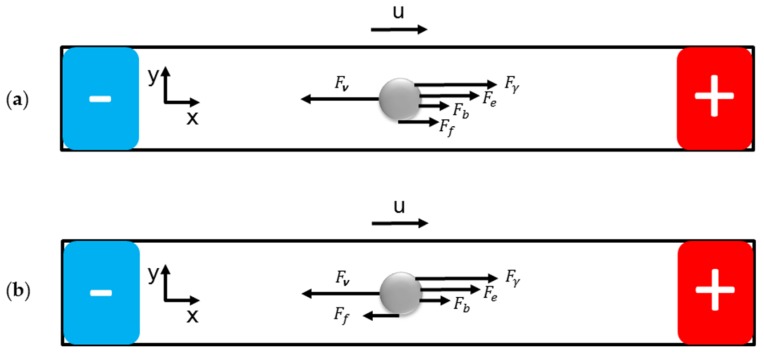
Forces analysis diagram for the locomotive liquid metal droplet in the electrical field when (**a**) the friction force $F_{f}$ is an impetus; (**b**) the friction force $F_{f}$ is a resistance.

There is a pressure difference between the two sides of the droplet, which generates the propulsive force $F_{\gamma }$ for the droplet to move right as Figure 2 presents.

In addition, there are another two driving forces actuating the droplet: the electric force $F_{e}$ and the bubble propelling force $F_{b}$. Hindrance is the force $F_{\nu }$ due to flow viscosity. Whether the friction force $F_{f}$ is an impetus or resistance remains unknown. If the droplet is regarded as a rigid ball, according to the Reynold number, the relative flow of the solution is laminar flow. The viscous force $F_{\nu }$ between the solution and the liquid metal droplet can be expressed as [36]:

(3) $$F_{\nu }=6\pi \mu uR$$

Considering gravity *G*, buoyancy $F_{be}$ from the electrolyte and buoyancy $F_{bb}$ from the bubble generated from the chemical reaction of aluminum and electrolyte on vertical direction, the rolling friction force $F_{f}$ is demonstrated as

(4) $$F_{f}=f\cdot \left(G-F_{be}-F_{bb}\right)=f\cdot g\cdot \frac{4}{3}\pi R^{3}\left(\rho_{m}-\rho_{a}\right)-f\cdot F_{bb}$$

where *f* is the rolling friction coefficient between the base of the channel and the droplet, g is the gravity acceleration, $\rho_{m}$ and $\rho_{a}$ are the densities of the liquid metal droplet and the electrolyte, $\rho_{m}=6.0165\times 10^{3}\;\mathrm{kg}/m^{3}$ and $\;\rho_{a}=10^{3}\;\mathrm{kg}/m^{3}$, *R* is the radius of the droplet. 

Deriving from the Navier–Stokes equation in the *x* direction, one has:

(5) $$\rho_{a}\left(\frac{\partial u}{\partial \tau }+u\frac{\partial u}{\partial x}+\nu \frac{\partial u}{\partial y}\right)=F_{x}-\frac{\partial p}{\partial x}+\rho_{a}\nu \left(\frac{\partial^{2}u}{\partial x^{2}}+\frac{\partial^{2}u}{\partial y^{2}}\right)$$

Regarding the model as steady-state laminar flow, ignoring the velocity in the *y* direction, the following momentum equation can be obtained:

(6) $$u\frac{\partial u}{\partial x}=\frac{F_{e}-F_{f}}{\frac{4}{3}\pi R^{3}\rho_{m}}-\frac{1}{\rho_{a}}\frac{dp}{dx}+\nu \left(\frac{\partial^{2}u}{\partial x^{2}}+\frac{\partial^{2}u}{\partial y^{2}}\right)$$

Extracting an infinitesimal Δx on the droplet, the pressure difference at two ends is

(7) $$\Delta p=\frac{\Delta S}{\Delta A}\cos\theta =\frac{\Delta S\cos\theta }{2\pi R\sin\theta \Delta x}$$

**Figure 3:**
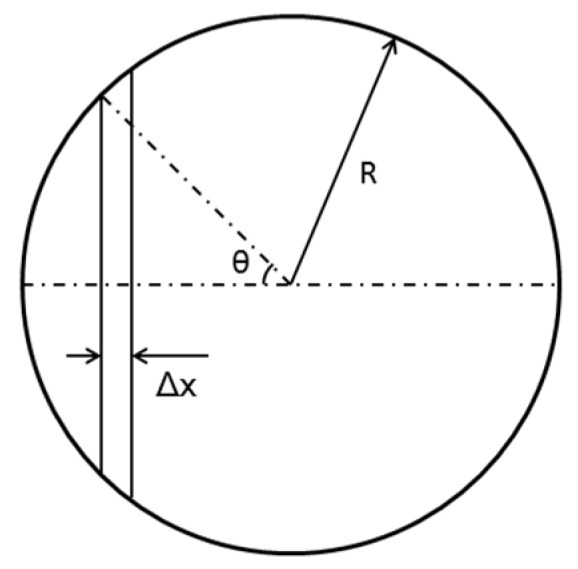
Geometrical meaning of parameters in equations.

Figure 3 shows the geometrical meaning of parameters in Equation (7).

Approximately, $\frac{dp}{dx}\approx \frac{\Delta p}{\Delta x}=\frac{2q_{0}\Delta \varphi }{R\Delta 2\pi R\tan\theta {\left(\Delta x\right)}^{2}}$. Thus,

(8) $$u\frac{\partial u}{\partial x}=\frac{q_{0}U}{\frac{4}{3}\pi R^{3}\rho_{m}L}-\frac{gf\left(\rho_{m}-\rho_{a}\right)}{\rho_{m}}-\frac{q_{0}\Delta \varphi }{\rho_{a}\pi R^{2}\tan\theta {\left(\Delta x\right)}^{2}}+\nu \left(\frac{\partial^{2}u}{\partial x^{2}}+\frac{\partial^{2}u}{\partial y^{2}}\right)$$

Taking the diameter of the droplet *D* as the length scale, *u* as the velocity scale, the voltage of the external electric field U as the electrical potential scale, Equation (8) can be non-dimensionalized as:

(9) $$\begin{array}{c} \frac{u^{2}}{D}\left(u^{*}\frac{\partial u^{*}}{\partial x^{*}}\right)=\frac{3q_{0}U}{4\pi D^{4}\rho_{m}}\frac{1}{{\left(R^{*}\right)}^{3}L^{*}}-\frac{gf\left(\rho_{m}-\rho_{a}\right)}{\rho_{m}}+\frac{q_{0}U}{\pi D^{4\rho_{a}}}\frac{\emptyset^{*}}{{\left(R^{*}\right)}^{2}\tan\theta {\left(\Delta x^{*}\right)}^{2}}\\ +\frac{\nu u}{D^{2}}\left(\frac{\partial^{2}u^{*}}{\partial x^{*}{}^{2}}+\frac{\partial^{2}u^{*}}{\partial y^{*}{}^{2}}\right) \end{array}$$

where $\emptyset^{*}=\frac{-\Delta \varphi }{U}$. The parameters with star-superscript are dimensionless. 

Divided by $\frac{\nu u}{D^{2}}$, Equation (9) can be converted into:

(10) $$\begin{array}{c} \frac{uD}{\nu }\left(u^{*}\frac{\partial u^{*}}{\partial x^{*}}\right)=\frac{3q_{0}U}{4\pi D^{2}\nu u\rho_{m}}\frac{1}{{\left(R^{*}\right)}^{3}L^{*}}-\frac{gf\left(\rho_{m}-\rho_{a}\right)D^{2}}{\rho_{m}u\nu }+\frac{q_{0}U}{\pi D^{2}\nu u\rho_{a}}\frac{\emptyset^{*}}{{\left(R^{*}\right)}^{2}\tan\theta {\left(\Delta x^{*}\right)}^{2}}\\ +\left(\frac{\partial^{2}u^{*}}{\partial x^{*}{}^{2}}+\frac{\partial^{2}u^{*}}{\partial y^{*}{}^{2}}\right) \end{array}$$

In Equation (10), the first combined quantity $\frac{uD}{\nu }$ is the Reynold number. $\left(u^{*}\frac{\partial u^{*}}{\partial x^{*}}\right)$, $\;\frac{1}{{\left(R^{*}\right)}^{3}L^{*}}$, $\frac{\emptyset^{*}}{{\left(R^{*}\right)}^{2}\tan\theta {\left(\Delta x^{*}\right)}^{2}}\;\mathrm{and}\;\left(\frac{\partial^{2}u^{*}}{\partial x^{*}{}^{2}}+\frac{\partial^{2}u^{*}}{\partial y^{*}{}^{2}}\right)$ are dimensionless terms, indicating that $\frac{3q_{0}U}{4\pi D^{2}\nu u\rho_{m}}+\frac{q_{0}U}{\pi D^{2}\nu u\rho_{a}}$ and $\;\frac{gf\left(\rho_{m}-\rho_{a}\right)D^{2}}{\rho_{m}u\nu }$ are non-dimensional. Respectively multiplied by the Reynold number $\;\frac{uD}{\nu }$, two new non-dimensional parameters are obtained as:

(11) $$Ä=\frac{q_{0}U}{\pi D\nu^{2}}\left(\frac{3}{4}\frac{1}{\rho_{m}}+\frac{1}{\rho_{a}}\right),\;\mathrm{and}$$

(12) $$Ö=\frac{gf\left(\rho_{m}-\rho_{a}\right)D^{3}}{\rho_{m}\nu^{2}}.$$

Physically, Ä is a metric of the ratio of the forces resulting from the electric field to the fluidic viscous force. The increase of Ä denotes that the forces induced by the electrical field strengthen. Ö is a metric of the ratio of the friction force to the fluidic viscous force.

### 3.2. The Voltage Effect

**Figure 4:**
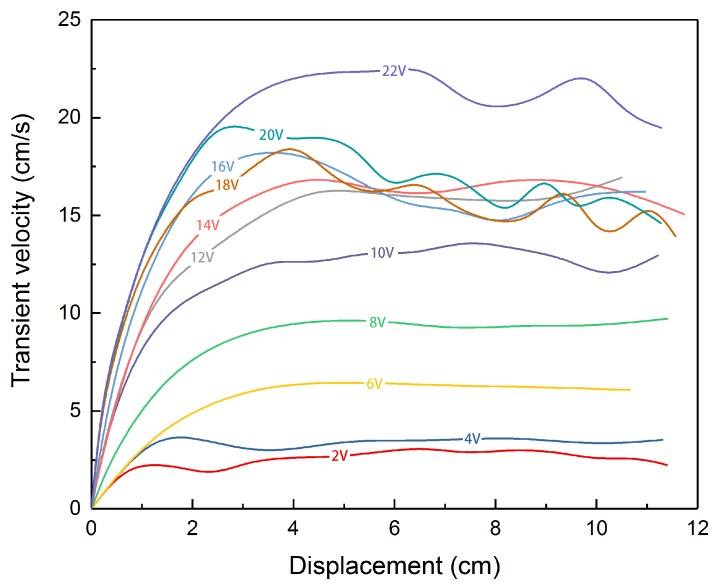
The transient velocity of the liquid metal droplet motor under different voltages.

The liquid metal droplet is injected statically at the cathode terminal initially. As the direct power supply is switched on, the droplet stretches and accelerates, then moves at a relatively steady speed towards the anode. When it approaches the anode terminal, it abruptly slows down and bounds back. Figure 4 shows the transient velocity of the liquid metal droplet under different voltages. We select the droplet with diameter of 2.5 mm and 0.2 mol/L sodium hydroxide solution from all the options. The applied voltages vary from 2 to 22 V, for water electrolysis intensifies as the voltage rises, creating more variables for the case when the voltage becomes too large. The channel is long and narrow, allowing the electrical field developed by two graphite rods to be approximately uniform. In the uniform electric field, the liquid metal droplet accelerates until the force reaches a balance and the droplet moves at a basically constant velocity. The curves tangle together when the applied voltage is above 10 V. The fluctuations of the curve, representing the oscillation of the motor during motion, can be drawn to the propelling bubbles generated by the liquid metal motor which consumes aluminum in advance, resulting in unpredictable moving direction. Plus, the inevitable adherence of the running droplet to the wall also intensifies the noise. 

**Figure 5:**
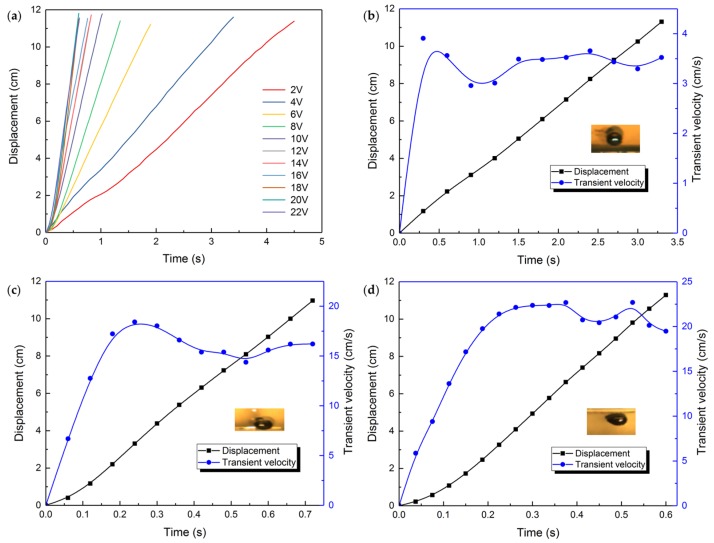
(**a**) Movement of the liquid metal droplet on a smooth surface under the impact of different voltages. The displacement and transient velocity change with time of the liquid metal droplet when the applied voltage is (**b**) 4 V; (**c**) 16 V; (**d**) 22 V, respectively, each with a snapshot of the droplet when it is moving at a constant speed.

The variations of the displacement and morphology with time are depicted in Figure 5. With low voltage, the droplet moves in a sphere without undergoing any noticeable deformation. As the voltage rises, both ends of the droplet deform differently, bringing about smaller curvature on the side with larger surface tension. When the voltage reaches 20 V, the deformation intensifies and a distinct pointy tail can be observed on the end opposite to the moving direction. Young–Laplace equation indicates that the surface tension difference is proportional to the potential difference. The increasing voltage magnifies the driving force stemmed from the surface tension difference on the liquid metal droplet. It should be noted that when the external voltage is 2 V or less, the electric force exerted on the liquid metal droplet is insufficient to overcome the resistance and the droplet would stagnate in the channel. 

### 3.3. The Size Effect

**Figure 6:**
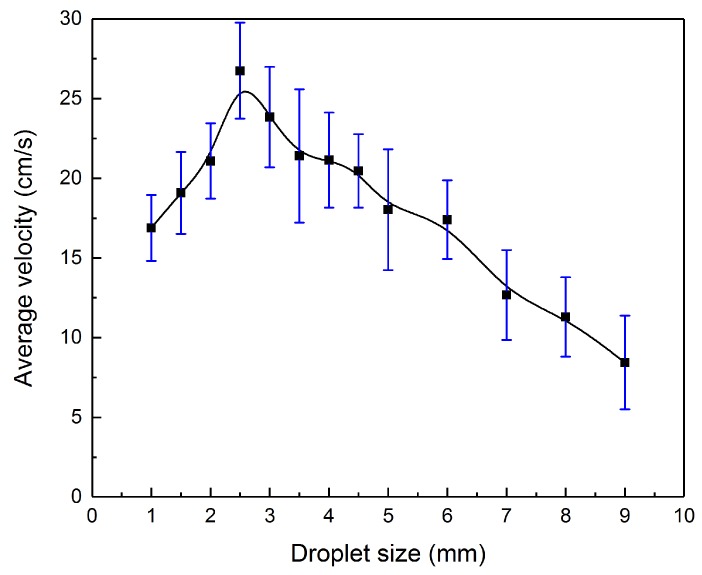
The average velocity of the motor varies with droplet sizes.

Figure 6 indicates how the average velocity of the motor varies with the droplet sizes from 1 to 9 mm under 20 V. The electrolyte solution remains 0.2 mol/L sodium hydroxide solution. Originally, the average velocity of the droplet rises with the magnified droplet size. As the diameter approaches 2.5 mm, the average velocity reaches the peak. Continuing to increase the droplet size, the velocity descends reversely. The surface tension driving force is positively correlative to the diameter of the droplet, as Equation (13) [30] shows:

(13) $$F_{\gamma }=\frac{U}{R_{t}}\frac{4\pi q_{0}\rho_{a}R^{2}}{h_{1}h_{2}-2\pi R^{2}/3}.$$

When the droplet size is small, the surface tension driving force $F_{\gamma }$ is too weak to actuate the droplet. Mounting the droplet size, the driving force will ascend and surpass the viscous and frictional drags. However, the viscous force $F_{\nu }$ between the solution and the liquid metal droplet increases linearly with the droplet size learned from Equation (2). As the droplet size enlarges, the viscous force gradually takes a dominant role in the net force. As a consequence, the average velocity declines as the droplet size climbs further. 

### 3.4. The Concentration Effect

**Figure 7:**
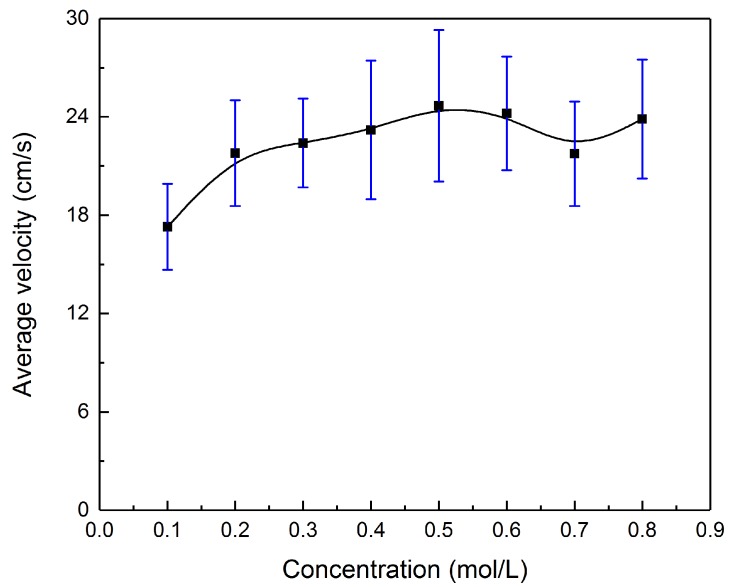
The average velocity of the motor varies with solution concentrations.

As illustrated in Figure 7, different solution concentrations also influence the locomotion performance of the liquid metal droplet. The diameter of the droplet is 2.5 mm and the applied voltage is 20 V. Surprisingly, the concentration has little impact on the average velocities of the droplets which are already moving at a constant speed but rather on the acceleration process. As can be seen from the curve, the average velocity of the droplet increases slowly at first with the rising concentration and attains the summit at about 0.5 mol/L, followed by a gentle fluctuation. The rising concentration enhances the electrical conductivity of the solution [37] and results in the shrink of the total electrical resistance. On the other hand, the initial EDL charge density $q_{0}$ rises as the concentration grows [30], accordingly strengthening the surface tension driving force $\;F_{\gamma }$. Therefore, the higher the concentration is, the faster the droplet runs. Nevertheless, the viscosity has an exponential relationship [38] with the concentration of the strong electrolyte solution, which would be a counter-balance when the droplet size goes up. Besides, when the concentration attains and exceeds 0.5 mol/L, the EDL charge density saturates. Therefore, the velocity flattens out.

### 3.5. The Friction Effect

**Figure 8:**
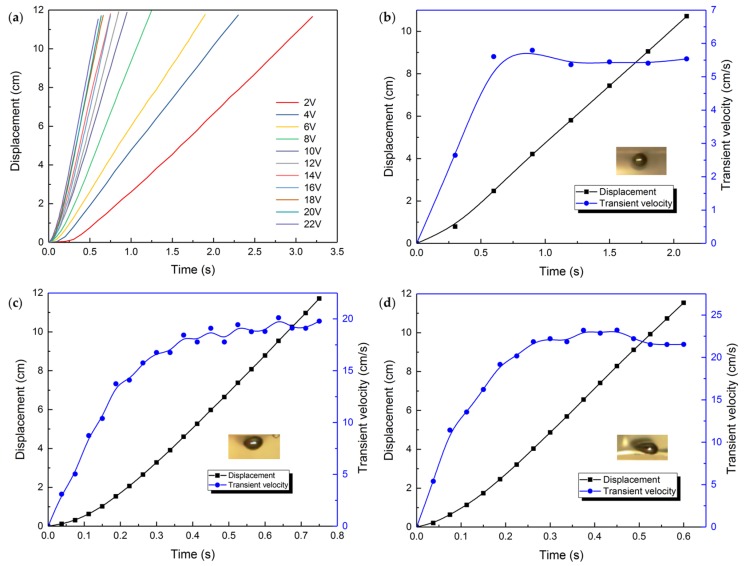
(**a**) Movement of the liquid metal droplet on a rough surface under the impact of different voltages. The displacement and transient velocity change with time of the liquid metal droplet when the applied voltage is (**b**) 4 V; (**c**) 16 V; (**d**) 22 V, respectively, each with a snapshot of the droplet when it is moving at a constant speed.

At last, after the above study, it still needs to verify that the friction between the bottom of the droplet and the sink base impelling the motion of the liquid metal motor instead of hindering. So we designed a contrast experiment accordingly, replacing the base from smooth glass to ground glass and maintaining other variables. The displacement and transformation tendency is shown in Figure 8. Little difference on deformation under the same voltage is observed. 

**Figure 9:**
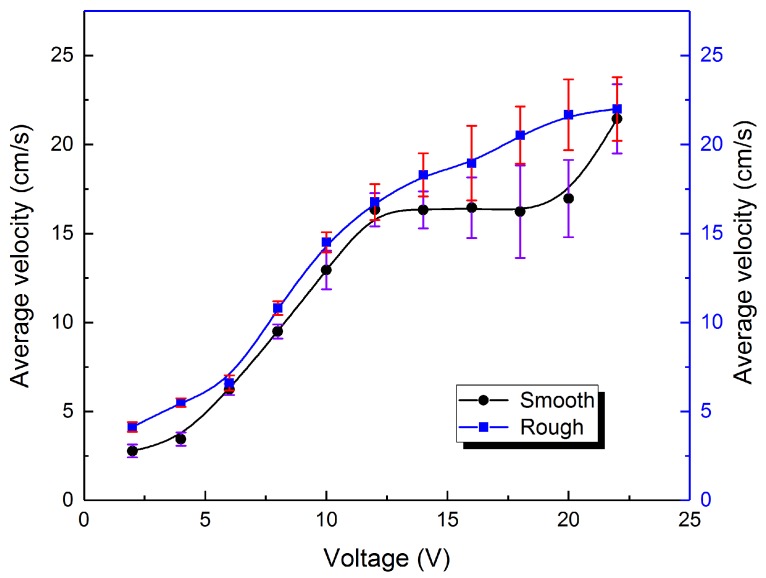
The comparison on average velocities of the liquid metal motor between the case of smooth base and the rough base.

The comparison on the average velocity of the liquid metal motor between the smooth base and the rough base can be seen from Figure 9. It is obvious that, with the increasing voltage, the average velocity of the liquid metal droplet rises gradually. The upward tendencies for two bases bear a close resemblance to each other, with the curve for the smooth base remaining underneath. The uncertainty grows with the velocity of the liquid metal droplet, on account of the existence of oscillation we mentioned earlier. Hence, it is safe to deduce that the friction given by the base of the groove drives the liquid metal droplet when it is moving towards the anode. Reasonable guess is that the contact of the bottom of the liquid metal and the surface of the groove base is close to the non-slip boundary condition. 

## 4. Conclusions

This work comprehensively disclosed the motion characteristics of liquid metal motors in the electric field, which is fed with aluminum in advance. Two new non-dimensional parameters, Ä and Ö, are proposed to evaluate the forces exerted on the droplet, representing the ratio of the forces resulting from the electric field to the fluidic viscous force and the ratio of the friction force to the fluidic viscous force respectively. Four essentials, electric field intensity, droplet size, solution concentration and surface roughness, are characterized based on compared experiments. The velocity of the droplet grows with the increasing applied voltage, yet the growth rate reducing. The average velocities of the liquid metal droplet go up and down as the sizes of the droplet increase, with the maximum velocity at the diameter around 2.5 mm. Concentration does not play a leading role in the moving process of the liquid metal motor, as the average velocity grows and flattens with the rising concentration. This work puts forward the idea that the friction between the bottom of the liquid metal and the surface of the groove base propels the droplet, implying the non-slip boundary condition on the liquid metal motor and the channel bottom interface. Further studies could focus on the numerical study of the inner flow field and deformation of the liquid metal motors. Coupled fields such as the combination of the magnetic field, the chemical field or the electric field might also give inspirations in the evolution of the precise control of the liquid metal, partially bringing the legendary science fiction to real life.
